# Construction of a reading literacy test item bank for fourth graders based on item response theory

**DOI:** 10.3389/fpsyg.2023.1103853

**Published:** 2023-05-25

**Authors:** Qishan Chen, Haiyan Zheng, Honglan Fan, Lei Mo

**Affiliations:** ^1^Philosophy and Social Science Laboratory of Reading and Development in Children and Adolescents (South China Normal University), Ministry of Education, Guangzhou, China; ^2^School of Psychology, South China Normal University, Guangzhou, China; ^3^College of Teacher Education, South China Normal University, Guangzhou, China

**Keywords:** reading literacy, item bank, item response theory, fourth grade, criterion-related validity

## Abstract

**Introduction:**

Reading literacy is not only central to students’ academic success during their school years but also crucial to their personal development in their later life. The development of assessment instruments for reading literacy has been of interest to researchers, educators and educational administrators. The purpose of the present study was to construct and validate a comparable item bank for assessing fourth-grade students’ reading literacy.

**Methods:**

One hundred fifteen reading comprehension items were developed and administered to 2,174 Grade 4 students to construct an item bank. Using the test equating technique and balanced incomplete block design, we divided participants into 10 subgroups, and the 115 items were further assigned into 10 test forms. Item response theory software was used to estimate discrimination, items’ threshold parameters, and students’ ability parameters. The criterion-related validity was also examined in 135 Grade 4 students who completed the reading literacy test and verbal self-description questionnaire.

**Results:**

The final item bank included 99 reading performance indicators to express high achievement. The correlation between the students’ reading literacy and the verbal self-description questionnaire was significant and demonstrated the item bank’s good criterion-related validity. The item bank developed in this study shows good psychometric characteristics and can be used to assess the reading literacy of fourth graders.

## Introduction

Reading and learning from text are some of the most complex and uniquely human cognitive activities. Consequently, the ability to read and comprehend text is not merely an essential element of school students’ academic achievement; it also plays a critical role in an individual’s personal development both at school and in his or her later life. Therefore, reading ability, also called reading literacy, is central and crucial for students’ academic success and is necessary for overall success in modern society.

Given the vital significance of reading literacy to an individual’s life, on the one hand, many researchers in psychology and linguistics (e.g., Kintsch, 1998; Alvermann et al., 2018; Kendeou and O’Brien, 2018) have paid attention to the cognitive processes of understanding, mental structures of reading representation, and numerous factors that contribute to successful text comprehension; moreover, these researchers have made numerous substantial achievements. On the other hand, over the years, many researchers (e.g., Mo, 1990, 1992, 1993; Wen, 2005; Song and Luo, 2018) and international organizations, such as the Organization for Economic Co-operation and Development (OECD) and the International Association for the Evaluation of Educational Achievement (IEA), have investigated and explored the construction and frameworks of reading literacy, administered some intranational or international programs/instruments to validate their theoretical hypotheses, or conducted numerous empirical studies. For instance, several programs, including the Programme for International Student Assessment (PISA) coordinated by the OECD; the Progress in International Reading Literacy Study (PIRLS), which is an international program conducted by IEA; the National Assessment of Educational Progress (NAEP), which constitutes a periodic assessment of student progress conducted in the United States by the National Center for Education Statistics, a division of the U.S. Department of Education, have been developed and administered to assess reading literacy worldwide.

Most of these programs are firmly focused on Western populations. Although these programs have been widely used and have firmly established their explicability in Western society, they have not been entirely suitable for assessing reading literacy among the Chinese population, and there have been relatively few empirical studies that have established the generalizability of these programs across societies outside the West and even fewer addressing traditional non-Western societies such as China.

The present study aimed at developing and validating a comparable item bank of reading literacy tests to evaluate fourth graders’ reading literacy performance in Chinese society. To achieve this purpose, a framework of reading literacy in Chinese society was proposed based on the assessment of reading literacy and reputable intranational or international programs of reading literacy. In particular, test items were evaluated using the modern psychometric approach and item response theory (IRT) to derive assessments from this research that are maximally reliable and valid.

### Modeling reading literacy

The purpose of this section is to propose and develop a working definition and assessment framework for reading literacy tests for Grade 4 students in China based on existing reading comprehension models and well-known reading measurement programs in conjunction with the definitions and requirements of *the Chinese Curriculum Standards for Full-time Compulsory Education*. In addition, this assessment framework is used as a guide for developing and screening test questions.

At least three issues must be taken into consideration in our assessment framework for modeling purposes. The first fundamental issue concerns the cognitive processing of reading comprehension. The second issue concerns the characteristics of reading materials. Lastly, the third issue for modeling reading literacy is the target percentage of assessment content.

#### The informational process of reading

Reading is the process by which readers obtain mental meaning from textual materials. The reading process involves complex cognitive processing activities, from word recognition and sentence comprehension to constructing discursive meaning. Many theoretical models of reading support that reading comprehension is a dynamic process in which readers process information explicitly stated in the text with the help of background knowledge, which causes mnemonic representations to change continuously, and these changing mental representations provide resources for the processing the next reading (Massey, 2014; Thiede and de Bruin, 2017; Alvermann et al., 2018; Kendeou and O’Brien, 2018). The theory of Kintsch (1998, 2018) suggests that the comprehension process is one in which the reader constructs a series of multilevel mental representations: surface code, text-based or proposition representation, and situation model. Surface code and text-based representation are mnemonic representations of the reading material’s words, concepts, propositions, and ideas. The situation model is a microcosm in which the reader interacts with his or her background knowledge based on text-based representations and forms a microcosm through reasoning. A situation model can be seen as a deep understanding of a text’s meaning.

Based on the scientific research on textual comprehension (Kintsch, 1998, 2018; Alvermann et al., 2018; Kendeou and O’Brien, 2018) and the definition of reading literacy emerging out of previous studies or programs—for instance, PISA (OECD, 2018, 2019), PIRLS (Mullis and Martin, 2019), NAEP (National Assessment Governing Board, 2019; Reilly et al., 2019), and the RAND Report (Snow, 2002)—the processes of comprehension in the present study were identified and classified as three fundamental elements: (a) retrieval and inference, (b) integration and interpretation, and (c) evaluation and reflection.

Retrieval and inference are based on the surface level of text representation and the independent parts of the texts, especially the explicit information included in the text. Here, inference refers to the straightforward inference based on the context and the information explicitly stated in the reading materials. For retrieval and inference tasks, the reader needs to identify textually explicit information and make simple inferences within and across texts; for example, the reader retrieves definitions, facts, and supporting details, locating specific information in the text, figurative language, topic sentence or main idea and identifying the author’s purpose and the causal relations, character traits, sequence of events or actions included in the text.

The integration and interpretation process, as in PISA’s or PIRLS’s frameworks, is based on the local and global meanings of texts. The process and the outcomes of integration and interpretation have been characterized by strong individuality. Reading tasks require the reader to make complex inferences within and across texts; for example, the reader summarizes central ideas, draws conclusions, finds evidence in support of an argument, determines unstated assumptions in an argument, infers mood or tone, integrates ideas to determine a theme, identifies or interprets a character’s motivations, and examines relations between themes and settings or characters.

Evaluating and reflecting require the reader to examine and evaluate the textual content, meaning, and textual features in terms of language and structure. For example, the reader judges the author’s craft and technique, evaluates the author’s perspective on the central topic, analyzes the presentation of information, judges the coherence or logic of an argument, and evaluates the way the author selects language to influence readers.

#### Characteristics of reading materials

Inspired by the prevision about the categories of reading materials in the PISA survey and the NAEP Reading Assessment, two-dimensional classifications (e.g., continuous texts vs. non-continuous texts and literary texts vs. informational texts) were considered in our framework. Following these criteria, four test forms were identified and labeled as continuous literary texts, non-continuous literary texts, continuous informational texts, and non-continuous informational texts. Moreover, there was no difference between continuous and non-continuous literary texts. Consequently, only three forms could be considered and identified in our reading literacy assessment framework.

The typical extensions for each text format could be categorized and described as follows: (1) literary texts include storytelling, fiction, literary nonfiction, poetry, narrative texts, and so on; (2) continuous informational texts include exposition and argumentation; (3) non-continuous informational texts include charts, graphs, tables, diagrams, forms, advertisements, and so on. Table 1 shows the assessment of the content of the reading process and the reading materials.

**Table 1:** The reading assessment framework for fourth graders based on the reading process and the format of reading materials.

Text format	Reading process		
	Retrieval and inference	Integration and interpretation	Evaluation and reflection
Literary texts	Author’s purpose; sequence of events or actions; identification of figurative language; character traits	Mood or tone inference; integration of ideas to determine a theme; interpretation of characters’ motivations; examination of relations between theme and setting or characters	Evaluation of the authors’ perspective about the main topic; evaluation of the way in which the authors select language to influence readers
Continuous informational texts	Definitions, topic sentence, supporting details, Location of specific information in texts, causal relations	Determination of unstated assumptions in an argument	Evaluation of the coherence or logic of an argument; evaluation of the authors’ craft and technique
Non-continuous informational texts	Facts, main idea	Summary of main ideas; drawing of conclusions; evidence findings in support of an argument	Analysis of information presentation

#### The target percentage of assessment content

The third fundamental issue for modeling reading literacy is the distribution and variety of texts that the students are asked to read for our test. Basically, this issue depends on the characteristics of the curriculum used by the fourth graders.

Based on the definitions and requirements of *the Chinese Curriculum Standards for Full-time Compulsory Education* and in conjunction with the actual situation of language teaching in schools and expert suggestions, we propose and develop the target percentage of assessment content based on the formats of reading materials. Specifically, 50% of the texts are literary texts, 25% are continuous informational texts, and 25% are non-continuous informational texts.

### Item response theory

Without accurate assessment, efforts to understand and ameliorate the definition and framework are unlikely to succeed. The measurement of psychological processes is not direct. Reading literacy is considered a latent trait, and a model is needed to describe the relationship between that latent trait and test-taking behaviors (Embretson and Reise, 2013). In general, previous attempts at constructing and developing reading comprehension tests have been conducted within the framework of classical test theory (CTT), which has been used in the Chinese context to test an individual’s reading ability or reading literacy (Mo, 1990, 1992; Wen, 2005). In the present study, the issue of reading literacy assessment is addressed within a different approach—the framework of IRT—to improve the quality of a comprehension test (Hambleton and Swaminathan, 2013; Min and Aryadoust, 2021).

IRT is a modern psychometric method widely applied in research in psychology, education, and linguistic. IRT has significant advantages over CTT in terms of instrumental development. The item parameters (e.g., item difficulty and discrimination) derived from CTT are overly dependent on the sample of participants on which the test is administered, resulting in limited generalizability. As a result, the tests developed are only applicable to a target population that is similar to that in the test sample. In addition, the difficulty of an item or a test and the examinees’ ability are defined based on two unrelated metric systems; therefore, their values cannot be compared. However, when developing tests based on IRT, the item parameters of the instrument (e.g., threshold parameters) and the examinees’ ability are estimated independently (Hambleton and Swaminathan, 2013). The relationship between examinees’ ability and item thresholds can be described using a monotonical function—the item characteristic curve (ICC)—which describes the relationship between changes in an examinee’s ability level and changes in the probability of a correct response. Even when examinees are administered two tests consisting of completely different item sets, the estimated levels of examinees’ traits will still take values on the same metric system and remain the same. That is, the estimation of an examinee’s ability is not dependent on a particular test item.

**Figure 1:**
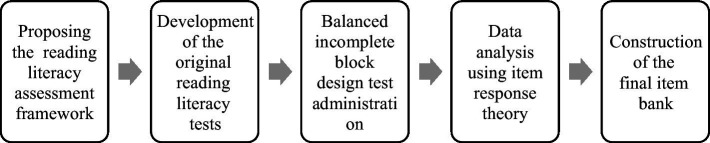
Study’s conceptual model.

Due to its psychometric properties, the IRT-based reading comprehension test may be a better measure of reading literacy than tests used in prior research. Therefore, the main purpose of this study is to develop an IRT-based comprehension test for future research. The conceptual model used in this study is as follows (Figure 1).

## Methods

### Participants

For sampling purposes, the population of Chinese fourth graders was divided into six strata based on the regions of China (North China, South China) and the average performance level of the school at stake (high level, medium level, and low level). A stratified cluster sample of schools was drawn according to the sampling plan. This sampling method allows the clusters within a stratum to be close in size and the differences between clusters to be small, while allowing stratified sampling to have small within-stratum differences and wide between-stratum differences, thus greatly improving the sampling effect (Li, 1994). A total of 2,193 questionnaires were collected, and 2,174 questionnaires were valid. Among the total sample of 2,174 fourth grader participants, there were 1,117 boys and 1,053 girls, and 4 did not report their gender. All participants were native Chinese speakers who could read Chinese fluently and did not suffer from reading dyslexia. They all had a normal or corrected-to-normal vision.

### Instrument development

The instrument development process was carried out in four phases, which are described below.

#### Phase 1: development of the original item bank

In this phase, the original item bank, which contained 34 texts and 206 test items, was prepared.

The original item bank was designed and developed by an expert panel. The panel consisted of 26 experts and reviewers who had extensive experience working in the practice of teaching reading and educational testing.

First, one group of experts, who were all Chinese teachers and Chinese teaching researchers, selected reading materials and developed test items according to our framework about reading literacy. To ensure the fitness of the reading material and the correctness of the test items, they were all analyzed by other groups of reviewers (at least 2–3 reviewers for each text and the items that accompanied it), all of whom were Chinese teachers or Chinese philologists. A number of mistakes were found and identified by the reviewers. Some of the mistakes were labeled as revisable mistakes, and some potentially incorrect test items, such as spelling or typographic errors, test items that had more than one possible correct answer, and violations of grammatical rules, were corrected and reviewed again. The reading materials or test items were removed if they were identified as incorrect or irrelevant or if the items contained any mistakes and were labeled as inadequate for revisions.

Afterward, a pilot test was conducted with Grade 4 subjects recruited in Guangzhou City. Another group of experts and reviewers, who were all Chinese teachers, Chinese philologists, and psychologists, were consulted to select the key and appropriate reading materials and test items for the main study, and the psychometric qualities of test items were defined in a pilot study. At that stage, four pieces of information for each item were considered and reviewed: the exclusivity of the correct answer, the linguistic subject of the item measured, the level of difficulty, and the effectiveness or discrimination of the item. Some mistakes were identified and corrected in that phase.

Finally, the original item bank retained 23 texts and 115 test items written in Chinese, and the text covered different types of literature and various topics. The mean number of Chinese characters per text was 578 (ranging in length from 95 to 922). Each text was accompanied by 4 to 6 four-option multiple-choice items.

#### Phase 2: calibration design

It was impossible to require all participants to answer all 115 items in the assessment. If students were required to spend several hours answering all items, on the one hand, it would negatively impact students’ participation rates. On the other hand, student performance would deteriorate due to the effect of fatigue and decreasing motivation in an extended time assessment. Therefore, in that phase, the remaining 115 items were distributed by following a balanced incomplete block design (BIBD). Ten booklets were defined, each of them containing 23 items, so that any participant could complete a booklet in less than 60 min.

The BIBD booklet design is a complex variant of matrix sampling, which is widely used in large-scale educational assessments and other similar surveys (Johnson, 1992; Van der Linden et al., 2004; Huang and Shih, 2021). The BIBD-spiraling design applied to NAEP is explained in Johnson’s (1992) paper. In short, the BIBD technique divides the total number of assessment items into small blocks, each of which is designed to take the same amount of time for completion. The blocks are assembled into booklets containing two or three blocks. The total set of booklets is spiraled and given to students, ensuring that every item block is administered to a representative sample of students. In BIBD, the blocks are assigned to booklets such that each block appears in the same number of booklets, and every block appears in at least one booklet, so the design is balanced. However, no booklet contains all items, so the data obtained from each assessed student are incomplete; the design is incomplete.

According to the BIBD-spiraling design, 115 items were divided into 10 blocks, which were further assigned into 10 booklets in a systematic sequence. As presented in Table 2, the spiral design ensured that each booklet appeared an appropriate number of times and ensured approximately equal numbers of student responses to each booklet. Furthermore, the spiral design presented each block of items to fewer students at any school but presented each block at more schools. The participants within the same test session were assigned booklets in the order in which the booklets were bundled. Therefore, each student in a test session received a specific booklet. Only a few students received the same booklet or block of items.

**Table 2:** Calibration design of reading literacy module administration.

Block	Booklet										*N* of valid records	*N* of items
	1	2	3	4	5	6	7	8	9	10		
1	√									√	434	13
2	√	√									451	10
3		√	√								450	13
4			√	√							443	10
5				√	√						435	13
6					√	√					436	10
7						√	√				436	13
8							√	√			428	10
9								√	√		419	13
10									√	√	416	10
*N* of records	225	226	224	219	216	220	216	212	207	209	2,174	
*N* of items	23	23	23	23	23	23	23	23	23	23		115

#### Phase 3: test administration

In that phase, the reading test booklets were administered with applied paper and pencil test forms along with a packet of demographic and informational measures by grouping tests to collect the responses of participants.

Participants were tested in groups during their regular class. First, all participants were given instructions about the general procedure of the investigation. Then, they were informed that they would read five texts on different topics and answer test questions for each text.

Afterward, the reading comprehension test booklets, attached with an identification code for each student on the first page, were passed out. Texts and question items were presented in booklet form, with the answer sheet appearing on separate pages from the texts and items. All participants were told that the purpose of the study was to assess how well they performed on a reading comprehension test. They were asked to carefully read the concrete instructions on the booklet’s first page. For instance, they were asked to read the texts carefully, and it was not recommended that the item be omitted for statistical purposes; it was recommended that the answer be modified clearly if the need arose. The reading time was self-paced by the examinee because test anxiety and rush had a negative effect on participants’ performance and parameter estimation. The participants were told they could take as much time as needed, but it was recommended to them that they use a maximum of 1 h to complete the whole reading test. Our pilot study showed that the interval was ample enough for more than 95% of the participants to finish the test.

An additional questionnaire was administered to a random sample of 135 participants from one school. After reading and comprehension testing, these participants were given a break. Then, they completed the verbal subscale of the self-description questionnaire (SDQ, Marsh et al., 2005).

#### Phase 4: data analysis

In that phase, the participants’ responses were collected, cleaned, included, and analyzed based on CTT and IRT. As a result, some items were removed from the item bank; the validity of the item bank was examined; and the discrimination parameters, threshold parameters of items, and ability parameters of students were estimated.

##### Previous analyses (identifying and removing unacceptable items from a psychometrical perspective)

Reading tests and questionnaire sessions were conducted by administering the booklet to 10 groups of fourth graders. A total of 2,193 booklets were completed, 2,174 of which were validated by experts in educational and psychological measurements according to validation criteria. Nineteen booklets were removed. Some of these booklets were identified as including anomalous response patterns, were deemed to be unacceptable from a psychometrical perspective, and were thus discarded from the input sample. For instance, the same option was selected for all test items.

##### Item response theory analyses (psychometric assessment and selection of comparable items)

The 10 reading test modules were calibrated using the two-parameter logistic item response model (2PLM) for dichotomous items. The 2PLM was written as


$$P_{g}\left(\theta \right)=P\left(X_{g}=1|\theta \right)=\frac{\exp\left[\alpha_{g}\left(\theta -\delta_{g}\right)\right]}{1+\exp\left[\alpha_{g}\left(\theta -\delta_{g}\right)\right]}$$

where $P_{g}\left(\theta \right)$ is the probability that an examinee with the ability θ (in this case, reading literacy) answers an item g correctly; $\alpha_{g}$ is the discrimination parameter indicating the degree to which small differences in ability are associated with different probabilities of correctly answering an item g, and $\delta_{g}$ is the threshold parameter corresponding to the ability level associated with a 0.50 probability of respondents answering the item g correctly. The 2PLM estimates item threshold and discrimination parameters, and it is assumed that the guessing parameter or the probability that an examinee who is infinitely low on the ability to answer an item g correctly is equal across items. We examined the fitness of the model and estimated the parameters using the BILOG-MG 3.0 program (Zimowski et al., 2003).

##### Validity analyses

After IRT analyses, the item bank’s criterion-related validity was also examined using the statistical software SPSS.

## Results

### Examination of uni-dimensionality

IRT assumes that examinees’ responses to items are related to a single underlying latent variable; therefore, determining whether a test violates the assumption of uni-dimensionality is a fundamental precondition to IRT analysis. Strict uni-dimensionality is unrealistic with any test instrument. However, the assumption of uni-dimensionality is generally considered satisfied if a single dominant factor is present among the test items, including one test (Embretson and Reise, 2013). Therefore, before the IRT analysis was conducted, the assumption of uni-dimensionality was first examined through the use of both exploratory factor analyses (EFAs). In this study, forced by the calibration design, the reading tests were conducted by administering the blocks of 10 booklets. Therefore, principal axis factor analyses with varimax rotation were performed separately for each booklet using SPSS software to determine whether one dominant first factor was run through the test items that were included in each booklet.

Evidence of a single dominant factor would emerge if the size of the eigenvalues associated with the first factor were larger than those associated with the second factor and if the first factor accounted for approximately 20% or more of the variance (Smith and Reise, 1998). The results showed that the ratio values of the eigenvalue between the first factor and the second-largest factor ranged in magnitudes from 1.11 to 1.82 for the booklets, and the eigenvalue of the second factor was similar to the other factors. The first factor accounted for an average of 19.28% of the variance. These results indicate that performance on the comprehension test for each booklet could be accounted for by a single dominant trait.

Additionally, the scree plot test, which is a widely used test of uni-dimensionality (Bentler and Yuan, 1998; Slocum-Gori et al., 2009), also showed a clear dominance of the first factor, which then dropped sharply to the level of the second and third factors for each booklet. This result was taken as further evidence that the items were indicators of a common latent factor.

### Model fit and item parameter estimation

IRT analysis was conducted for all the remaining 115 items. The item discrimination parameter (*a*) and item threshold parameter (*b*) were estimated using the 2PLM.

IRT analysis was conducted in a stepwise manner for the total sample. The item parameters for all items could be computed using the software BILOG-MG 3.0. The software BILOG provided chi-square item-fit statistics, which could assess the fit of individual test items. These item-fit statistics were derived by sorting examinees into groups based on their trait level and comparing the observed proportion endorsed within a group with that predicted by the estimated ICC. To achieve satisfactory goodness-of-fit indexes of the 2PLM, 16 items were eliminated, and 99 items were remained.

#### Item threshold

Negative *b* parameter values revealed that the items were easy, which meant that they could be answered correctly even by individuals with low trait levels, whereas positive *b* parameter values indicated that the items were difficult, which meant that they could be answered correctly only by individuals with high trait levels. As seen in Table 2, the item threshold estimates ranged from −3.81 to 3.34. The obtained average values for the *b* parameter were negative (*M* = −1.130, *SD* = 1.43), which meant that the items in the bank were generally easy.

#### Item discrimination

The *a* parameter values ranged from 0.14 to 1.12 (*M* = 0.50, *SD* = 0.20). This finding indicates that the items on the comprehension test were moderate.

#### Ability estimation

Students’ ability levels ranged from −3.04 to 1.89 (*M* = 0.13, *SD* = 0.81).

### Validity analyses

The criterion-related validity was analyzed by calculating the correlation coefficient between the reading literacy score and the verbal SDQ score. The Cronbach’s alpha of the SDQ in this sample (*N* = 135) was 0.88. The correlation coefficient of the students’ reading literacy and SDQ verbal scores was 0.32, and the *p-*value was below 0.001. A moderate but statistically significant correlation demonstrated a good criterion-related validity of the item bank developed in this study.

## Discussion

The purpose of the present study was to develop and validate a comparable item bank of reading literacy tests for evaluating fourth graders’ reading comprehension performance in Chinese society based on IRT. Unlike the PISA, PIRLS, and NAEP, which mostly focus on Western populations, the new instrument includes texts and items that evaluate the subjects’ reading comprehension ability in traditionally non-Western societies such as China. The results show that the instrument has good validity and reliability.

Most of the reading literacy measures in the empirical literature have relied on the CTT and generally have not taken full advantage of IRT analysis in the scale development process (Mo, 1990, 1992, 1993; Wen, 2005). The potential advantage of utilizing IRT analysis in instrument development is that IRT provides greater flexibility in selecting items from the existing item bank tailored to the objectives of a particular research investigation.

The issues concerning the processes of item bank development and administration, such as calibration design, item selection rules, test validity, and scoring methods, are well documented in the literature (Johnson, 1992; Van der Linden et al., 2004; Embretson and Reise, 2013; Hambleton and Swaminathan, 2013; Min and Aryadoust, 2021). The typical process of item bank development is as follows. First, the original item bank is developed and divided into several subtests to collect the responses from representative samples. Then, the subtest scores and item parameters are statistically analyzed and estimated. Finally, the final item bank is made to have a common scale by equating.

This paper has presented in detail the process by which a reading literacy test bank has been developed based on an iterative series of IRT analyses that consider scale dimensionality and item parameters. As a prerequisite assumption for the IRT analysis, the uni-dimensionality of the items used to develop the item bank was verified. The results of the item fit testing showed that 16 items that did not reach a satisfactory level in the 2PLM goodness-to-fit indexes were removed. The remaining 99 items were then estimated for the *b* parameter and *a* parameter, and it was found that the obtained average values for the *b* parameter were negative, which meant that the items were generally easy. Moreover, most of the values of the *a* parameter were at a medium level, which indicates that these items have medium item discrimination for students’ reading ability. In sum, the comprehension test should be helpful for distinguishing participants, especially those with lower levels of ability. In other words, it would be most appropriate to use the comprehension test when the target population is at the low end of the ability continuum.

This study also conducted a validity analysis of the remaining items, and the results indicated that these items were valid for assessing students’ reading ability. A final item bank of 99 items was generated and created from 23 textual sources of reading.

The average number of characters in these 23 reading sources was 499 (ranging from 37 to 875), and these sources included literary texts (e.g., fiction, literary nonfiction, poetry, narrative text, and so on), continuous informational texts (e.g., exposition text, argumentation text, and so on), and non-continuous informational texts (e.g., chart, argumentation text, and so on).

### Theoretical and practical implications

The findings of the present study contribute to the literature on theoretical models and measures of reading literacy. This study combines well-known reading comprehension theories, the assessment frameworks of large-scale educational assessments and *the Chinese Curriculum Standards for Full-time Compulsory Education* to develop an item bank to measure fourth graders’ reading literacy. This study is an attempt to place Western reading theories and assessment frameworks in the Chinese cultural context, providing evidence for the cross-cultural adaptability of reading comprehension models and international educational assessment frameworks, as well as reflecting on Chinese characteristics. This study is based on the IRT approach; the study uses a 2PLM for parameter estimation and rigorous screening of test items based on the results of parameter estimation, which provides a more objective and accurate test of students’ reading literacy and provides a theoretical approach and implementation path that can be used for the development of similar reading literacy tests in the future.

The results of this study have important practical implications for the assessment and development of reading literacy in China. The item bank developed in this study can be used for general language testing in Chinese schools and can also be provided to educational administration agencies for the assessment and monitoring of the educational quality of reading literacy. The items developed in this study present a number of advantages beyond those currently used in Chinese schools, such as the characteristics of the reading materials and the psychometric characteristics of the item. This approach can be used to measure students’ reading literacy more accurately on the one hand and to “promote teaching with testing” on the other hand, which can feed into the teaching of reading and improve the effectiveness of education, contributing to improvements in China’s compulsory education. The assessment and monitoring of compulsory educational quality is an important issue that benefits the country and the people, and accurate assessment and monitoring require scientific instruments. The test items developed in this study have suitable psychometric characteristics and can be used directly or modified to assess the quality of compulsory education. The procedures and methods used in developing the item bank in this study can also be used to inform practical work on developing assessment instruments for educational quality assessment and monitoring.

### Limitations and future research

Although this study has developed a valuable item bank, several limitations need to be addressed in future research. First, we could not conduct a factor analysis across the entire item bank when testing uni-dimensionality because the original item bank was divided into 10 booklets. Although the reading items were randomly assigned to test blocks and thus combined into different booklets, which can be viewed as replications, we still could not ensure that reading items from different booklets did not exhibit the prerequisite requirement of local dependence. Future research could attempt to create additional types of booklets by combining the various test blocks in different ways to better test the uni-dimensionality of the items within the entire item bank. Second, socioeconomic and technological developments have made mobile phones and computers into an integral part of our lives, and the reading of electronic texts has been integrated into every aspect of our studying, working, and living. Reading literacy is not only the key to opening up the world of traditional print texts but is also vital for electronic texts. The reading comprehension processes and reading strategies required to read print and electronic texts are both similar to and different from each other. Reading electronic texts requires readers to use a number of new reading strategies. For example, sifting through the vast amount of information on the internet to find the material needed accurately and quickly, comparative processing of multiple materials and critical thinking are particularly important. Our study did not cover electronic texts, and future research could attempt to incorporate electronic reading ability into the assessment framework and develop appropriate items to measure that ability.

## Conclusion

Overall, it is feasible to use different reading materials, such as literary texts, continuous informational texts, and non-continuous informational texts, to measure the reading ability of Grade 4 students at three levels (e.g., retrieval and inference, integration and interpretation, and evaluation and reflection). The item bank developed in this study contains 23 texts with 99 test items. It exhibits appropriate psychometric characteristics in terms of difficulty, discrimination and validity for the assessment of reading literacy in Grade 4 students.

## Data availability statement

The raw data supporting the conclusions of this article will be made available by the authors, without undue reservation.

## Ethics statement

The studies involving human participants were reviewed and approved by the Research Ethics Committee of the South China Normal University. Written informed consent to participate in this study was provided by the participants’ legal guardian/next of kin.

## Author contributions

QC, HZ, HF, and LM made a significant contribution to the work reported, whether that is in the conception, study design, execution, acquisition of data, analysis and interpretation, or in all these areas; took part in drafting, revising or critically reviewing the article; gave final approval of the version to be published; have agreed on the journal to which the article has been submitted; and agree to be accountable for all aspects of the work. All authors contributed to the article and approved the submitted version.

## Funding

This research was supported by the Research Project of Philosophy and Social Science in Guangdong, China (GD22CXL01) and the Educational Science Planning Project (Higher Education Special Project) in Guangdong Province, China (2022GXJK176).

## Conflict of interest

The authors declare that the research was conducted in the absence of any commercial or financial relationships that could be construed as a potential conflict of interest.

## Publisher’s note

All claims expressed in this article are solely those of the authors and do not necessarily represent those of their affiliated organizations, or those of the publisher, the editors and the reviewers. Any product that may be evaluated in this article, or claim that may be made by its manufacturer, is not guaranteed or endorsed by the publisher.
